# Apoptosis and its pathways as targets for intracellular pathogens to persist in cells

**DOI:** 10.1007/s00436-023-08031-x

**Published:** 2023-12-19

**Authors:** Jorge Rodríguez-González, Laila Gutiérrez-Kobeh

**Affiliations:** 1 Unidad de Investigación UNAM-INC, División de Investigación, Facultad de Medicina, Universidad Nacional Autónoma de México-Instituto Nacional de Cardiología “Ignacio Chávez,”, Juan Badiano No. 1, Col. Belisario Domínguez, Sección XVI, Delegación Tlalpan, C.P. 14080 Ciudad de México, México; 2 Present Address: Laboratorio de Estudios Epidemiológicos, Clínicos, Diseños Experimentales e Investigación, Facultad de Ciencias Químicas, Universidad Autónoma “Benito Juárez” de Oaxaca, Oaxaca, Mexico

**Keywords:** Apoptosis, Bacteria, Fungi, Intracellular pathogens, Parasites, Signaling pathways

## Abstract

Apoptosis is a finely programmed process of cell death in which cells silently dismantle and actively participate in several operations such as immune response, differentiation, and cell growth. It can be initiated by three main pathways: the extrinsic, the perforin granzyme, and the intrinsic that culminate in the activation of several proteins in charge of tearing down the cell. On the other hand, apoptosis represents an ordeal for pathogens that live inside cells and maintain a strong dependency with them; thus, they have evolved multiple strategies to manipulate host cell apoptosis on their behalf. It has been widely documented that diverse intracellular bacteria, fungi, and parasites can interfere with most steps of the host cell apoptotic machinery to inhibit or induce apoptosis. Indeed, the inhibition of apoptosis is considered a virulence property shared by many intracellular pathogens to ensure productive replication. Some pathogens intervene at an early stage by interfering with the sensing of extracellular signals or transduction pathways. Others sense cellular stress or target the apoptosis regulator proteins of the Bcl-2 family or caspases. In many cases, the exact molecular mechanisms leading to the interference with the host cell apoptotic cascade are still unknown. However, intense research has been conducted to elucidate the strategies employed by intracellular pathogens to modulate host cell death. In this review, we summarize the main routes of activation of apoptosis and present several processes used by different bacteria, fungi, and parasites to modulate the apoptosis of their host cells.

## Introduction

Human health is continuously threatened by pathogens and in response to this, the immune system orchestrates multifaceted defense strategies. As a result, pathogens have developed multiple schemes to overcome human defense mechanisms. Among these, cell death is very important for the host because it eliminates infected cells and activates the immune system. However, cell death can become a double-edged sword: if uncontrolled, it may also lead to tissue damage, which might subsequently activate the pathogen dissemination procedures. To avoid this, the major cell death pathways (apoptosis, pyroptosis, and necroptosis) are highly regulated. Recently, these three programed cell death pathways have been included in an entity named PANoptosis directed by a PANoptosome. The PANoptosome is a multimeric protein complex responsible for recruiting all the molecules necessary to carry out any of the three types of programmed cell death (Bedoui et al. 2020; D'Arcy 2019; Samir et al. 2020). Of these three types of programmed cell death, apoptosis has typically been described through morphological markers such as chromatin condensation, cytoplasm contraction, formation and separation of apoptotic bodies, and the lack of inflammation. Nevertheless, the Committee for Nomenclature of Cell Death has determined that to have a clearer characterization of the different types of cell death quantifiable biochemical parameters must be used instead of morphological ones (Galluzzi et al. 2014).

### Apoptosis activation phases

Apoptosis transits through two main phases: the first one is the initiation phase that consists in the activation of genes and signaling pathways required for apoptosis. The second one is the executioner phase where the aspartate-specific peptidases, dependent on cysteine (caspases) 3, 6, and 7 participate after being, at the same time, activated by the initiation caspases 2, 8, 9, and 10. Upon activation, the executioner caspases are the enzymes in charge of dismantling the cell and provoking the morphological changes characteristic of apoptosis (Taylor et al. 2008).

Also, depending on the initiation phase, apoptosis has been divided as follows: (1) The extrinsic pathway or activated by receptors, (2) the perforin/granzyme pathway, and (3) the intrinsic or mitochondrial pathway. The extrinsic pathway is activated by extracellular processes through soluble ligands that bind to their corresponding receptors such as Fas/FasL associated with death domains. The perforin/granzyme pathway is crucial for the cytotoxic activity of cells of the immune system, such as NK cells and cytotoxic T lymphocytes. The intrinsic pathway is mainly induced by cellular stress such as DNA damage or the accumulation of misfolded proteins and greatly depends on mitochondrial activity. Nevertheless, dividing apoptosis according to the form of initiation is purely didactic since the pathways are not mutually exclusive and can activate each other through positive feedback circuits. Additionally, all routes converge in the executioner phase that is initiated with the activation of procaspase 3 and results in DNA fragmentation, nuclear and cytoskeleton proteins degradation, expression of ligands for phagocytic cells receptors, formation of apoptotic bodies, and finally phagocytosis by neighbor cells (Elmore 2007; Kashyap et al. 2021).

### The extrinsic pathway

As already mentioned, those in charge of starting this pathway are receptors encoded in the genes of the tumor necrosis factor receptor superfamily (TNFR) characterized by the presence of an intracellular domain of approximately 80 amino acid residues called death domain (DD). The best researched members of this family are the Fas receptor and its ligand FasL (FasR/FasL) and the tumor necrosis factor α1 and its ligand TNF-α (TNF-α/TNFR1). Other well-known members of this family are Apo3L/DR3, TRAIL/TRAILR, and netrin receptors (UNC5A-D). Interestingly, these last ones can only induce apoptosis when the specific ligand concentration drops below the critical point (Elmore 2007; Green and Llambi 2015). This pathway initiates when FasL binds FasR, which is an unstable homotrimer, stabilizes it and provokes conformational changes in the DD. Then, proapoptotic proteins such as FADD (DD union protein associated to FAS) are mobilized and associate through the death effector domain (DED) with the initiation procaspases 8 and 10, mainly 8. This association permits the autocatalytical activation of procaspase 8 or 10. These active caspases have as main substrates the executioner caspases 3, 6, and 7, most commonly 3. Lastly, active executioner caspases are responsible for the turnover of the cell. Interestingly, caspase 8 is a connection point between the extrinsic and the intrinsic pathway since it can also initiate the intrinsic pathway of apoptosis through the degradation of the pro-apoptotic protein Bid to form truncated Bid (tBid) that facilitates the release of apoptogenic proteins like cytochrome C (Elmore 2007; Green and Llambi 2015; Kashyap et al. 2021; Redza-Dutordoir and Averill-Bates 2016).

As just mentioned, TNF-α/TNFR are other important participants of the extrinsic pathway of apoptosis. Upon binding of TNF-α to TNFR, the receptor becomes a stable homotrimer that recruits TRADD (protein with death domain associated to TNFR). Then, TRADD recruits both RIP and TRAF and forms the complex I (Green and Llambi 2015). Additionally, TRAF2 can also recruit apoptosis inhibitory proteins (c-IAP) 1 and 2 (Rothe et al. 1995). c-IAP 1/2 ubiquitinate RIP kinase, which permits the activation of the NF-κB signaling pathway and cell survival (Varfolomeev et al. 2008). On the other hand, the inhibition of c-IAP 1/2 blocks RIP ubiquitination, which releases TRADD-RIP (complex II) from TNFR. Once in the cytoplasm, complex II recruits FADD, which in turn binds procaspase 8, activates it, and turns on subsequent executioner caspases (Elmore 2007; Green and Llambi 2015; Kashyap et al. 2021; Redza-Dutordoir and Averill-Bates 2016).

### The perforin/granzyme pathway

This pathway is crucial for cytotoxic cells of the immune system such as NK cells or cytotoxic T lymphocytes. Apoptosis is fundamental for these cells since they have the important task of eliminating aberrant cells such as tumor cells or cells infected with viruses or intracellular parasites. Once a cytotoxic cell senses a target cell, induces secretory lysosome exocytosis and the release of the cytotoxic contents of this organelle. Perforin form pores in the target cell membrane and facilitates the entry of the serine proteases named granzymes A and B into the target cell cytoplasm. Granzyme A can initiate DNA fragmentation independently of caspases. It degrades a nucleosome protein complex called SET, which inhibits NM23-H1 gene, and initiates DNAase NM23-H1 synthesis. For its part, granzyme B initiates apoptosis by several routes. It can cleave and directly activate procaspase 3, it can initiate the activation caspase cascade to reach the executioner caspases (3, 6, and 7) through the activation of procaspase 10, and finally can degrade the inhibitor of caspase activated DNAase (ICAD). Interestingly, granzyme B is also a point of connection with the intrinsic pathway since it can cleave Bid to form tBid and initiate apoptosis through this pathway (Elmore 2007).

### The intrinsic pathway

The medullary condition to initiate apoptosis through the mitochondrial or intrinsic pathway is cellular stress, which can be caused by different sources such as DNA damage, oxidative stress, accumulation of misfolded proteins in the endoplasmic reticulum, radiation, hypoxia, and nutrient deprivation. Notwithstanding the origin of the inducement, this pathway directs to a crucial step in apoptosis that is the mitochondrial outer membrane permeabilization (MOMP), where the proapoptotic proteins Bak and Bax play a leading role through their polymerization and formation of pores in the mitochondrial external membrane. Once this process has been triggered, it constitutes a point of no return in the apoptotic process due to the occurrence of several deadly events for the cell. These include as follows: (1) fading of ATP synthesis and active transport systems due to the dissipation of the membrane potential. (2) Liberation from the mitochondrial intermembrane to the cytoplasm space of distinct proteins such as cytochrome C, apoptosis inducing factor (AIF), endonuclease G (EndoG), direct union protein to IAP with low pI (DIABLO or SMAC), and high temperature requiring protein A2 (HTRA2) that are toxic for the cell. (3) Prevention of the respiratory chain. Once released to the cytoplasm, cytochrome C binds to the adaptor protein APAF1, activates it, and starts the formation of a multiproteic complex named apoptosome. This complex is a crucial hub in the induction of apoptosis since it recruits procaspase 9 that is activated by auto-proteolysis, which in turn activates procaspase 3 and initiates the cellular changes triggered by at least 1000 proteins processed by the downstream effector caspases (Crawford and Wells 2011; Galluzzi et al. 2012). Alternately, other molecules intervene in the dismantling of the cell independently of caspases. Such is the case of AIF and EndoG that translocate to the nucleus where they initiate fragmentation of DNA in segments of 50-300 kb carrying out apoptosis independently of caspases. Additionally, SMAC/DIABLO and HTRA2 inhibit the antiapoptotic functions of IAPs. Also, HTRA2 has serine protease activity, which contributes to the executioner phase of apoptosis independently of caspases (Elmore 2007; Galluzzi et al. 2012; Kashyap et al. 2021).

### Proteins that regulate MOMP

As already mentioned, MOMP is a no return step in apoptosis, thus its regulation is transcendental for the correct development of this important process. The proteins that are in charge of this crucial task belong to the Bcl-2 family of proteins where some have pro-apoptotic functions and other anti-apoptotic. This family of proteins is characterized for the homology domains called BH ranging from 1 to 4. The anti-apoptotic proteins possess 4 BH homology domains and members of this family are Bcl-2, Bcl-xL, MCL-1, A1, among others. Conversely, proteins with pro-apoptotic activity are subdivided in two groups. One group possesses homology domains BH1 to BH3 and includes the effectors Bax and Bak that polymerize in the mitochondrial membrane and initiate the MOMP. The second group solely possesses the homology domain BH3 such as Bad, Bid, Bik, Bmf, PUMA, NOXA, and Hrk. They sense apoptotic signals and activate Bax/Bak or inhibit Bcl-2 anti-apoptotic proteins. As previously noted, the cleavage of Bid, performed by caspase 8 and granzyme B, to form tBid is a pivotal point in apoptosis through which the granzyme/perforin and the extrinsic pathways are amplified by the intrinsic pathway (Dai et al. 2016; Kollek et al. 2016). The delicate balance between pro-apoptotic and anti-apoptotic proteins can determine the fate of the cells in such a way that an increase in the anti-apoptotic proteins can inhibit apoptosis or vice versa (Kale et al. 2018).

### Modulation of apoptosis by intracellular microorganisms

Intracellular microorganisms face a difficult task. On one hand, they need to invade their host cells in order to survive, but once inside, they have to dodge host defense mechanisms to avoid being eliminated. One important host defense mechanism is apoptosis, whose fine modulation is critical for intracellular pathogens to survive in the host. Thus, many pathogens have evolved strategies to both induce or inhibit apoptosis depending on the cellular context. One recurrent approach for the induction of apoptosis is the inhibition of innate immune signaling that blocks cytokine and anti-microbial effector production, and triggers cell death. Apparently, this mechanism of induction of apoptosis by intracellular microorganisms has been conserved through the course of evolution since it avoids the elimination by host antimicrobial effector molecules and allows the invasion of other cells or the acquisition of nutrients. On the other hand, under normal conditions, the sole presence and propagation of intracellular pathogens cause enormous cellular stress that induces apoptosis. Thus, successful colonization depends on the ability of intracellular pathogens to block apoptosis and to safeguard replicative niches and infect new neighbor cells.

### Modulation of the extrinsic pathway by intracellular pathogens

#### Modulation of extracellular signals

**Fig. 1:**
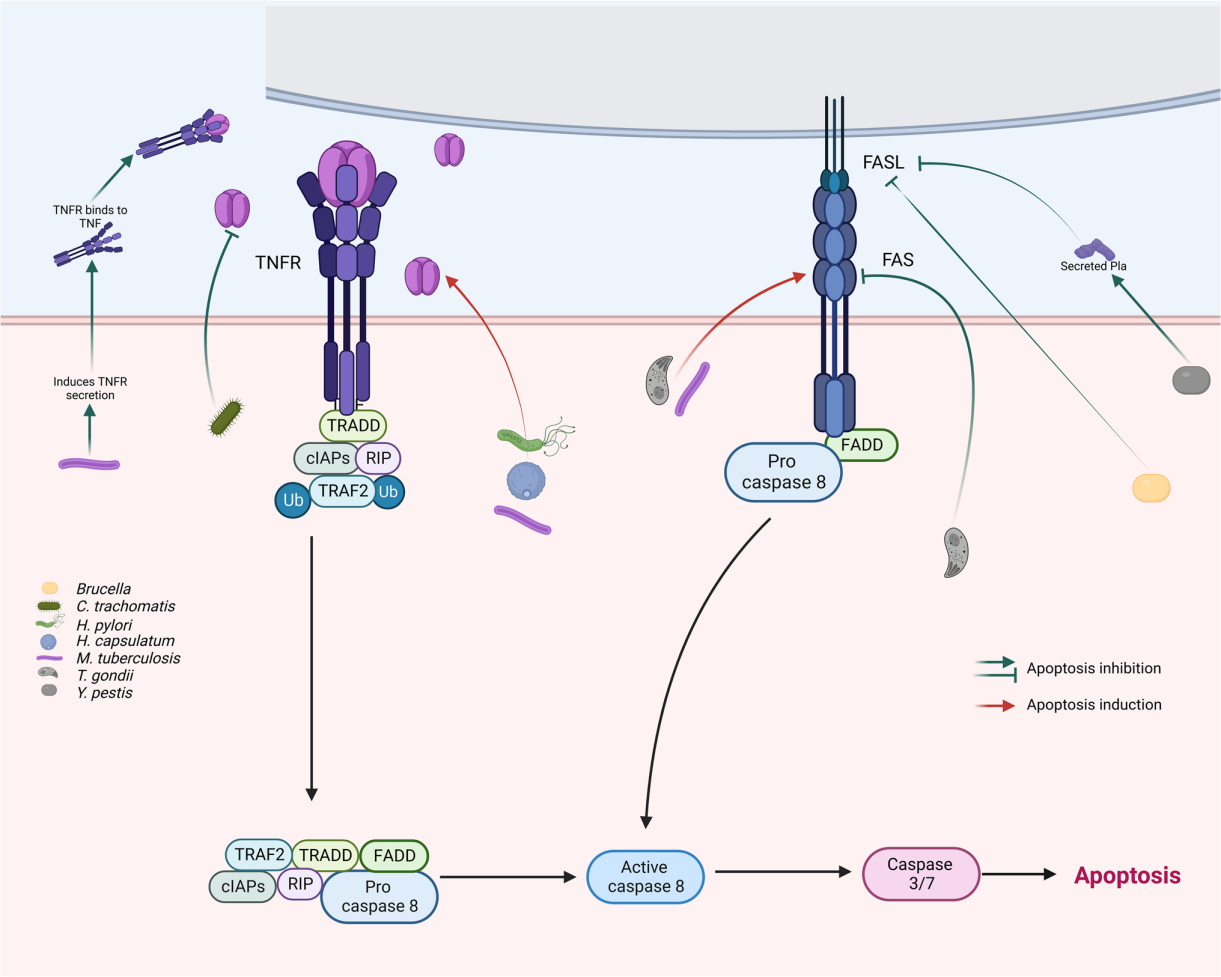
Modulation of the extrinsic pathway by intracellular pathogens. The induction of apoptosis through the extrinsic pathway occurs through the activation of death receptors of the TNF family such as TNFR or FAS. When death receptors bind to their ligands, adaptor proteins, such as TRADD or FADD, are recruited to the receptor intracellular domain and, in turn, recruit other proteins to form multiproteic complexes that activate initiation caspases, mainly caspase 8. Caspase 8 activates executioner caspases, mainly caspase 3, which finally conducts to cell apoptosis. Some intracellular pathogens modulate the extrinsic pathway of apoptosis. For example, *C*. *trachomatis* and *Salmonella* inhibit host cell apoptosis through the inhibition of TNFR or TNF, respectively. It is also known that *Brucella*, *M*. *tuberculosis*, *T*. *gondii*, and *Y*. *pestis* block host cell apoptosis through the inhibition of FAS or FASL. However, and in a very interesting way, it is known that *M*. *tuberculosis* and *T*. *gondii* can induce host cell apoptosis by enhancing TNF secretion or FASL expression. Besides, it has been observed that *M*. *tuberculosis* can also induce the expression of TNFR in order to capture the extracellular TNF and in this way inhibits apoptosis

The key participants of the extrinsic pathway of apoptosis are surface receptors that upon binding to their corresponding ligands unchain transduction pathways that culminate in the dismantling of the cells. Thus, it is not surprising that these extracellular signals or transduction pathways are important targets for extracellular pathogens to modulate apoptosis. Interestingly, also some intracellular pathogens can inhibit apoptosis during the establishment of infection by either blocking, inactivating or degrading key components of this apoptosis pathway. Due to the major role of Fas/FasL in the initiation of the extrinsic pathway, it is not remarkable that it is one of the main targets for intracellular pathogens to block apoptosis. A clear example of this is *Yersinia pestis*, a Gram-negative, non-motile, coccobacillus bacterium, that causes pneumonic plague. This bacterium abrogates the induction of FasL-mediated apoptosis and the activation of caspase-3/7 through the secretion of the plasminogen activator protease, Pla (Fig. 1). This protease degrades both the membrane-bound variant of FasL and soluble FasL (sFasL) shed from host cell surfaces. Pla also affects the FasL-dependent production of several inflammatory cytokines during pneumonic plague that is detrimental for the host against bacterial growth (Caulfield et al. 2014) Other interesting strategies directed toward Fas/FasL to avoid the induction of apoptosis through the extrinsic pathway are displayed by *Mycobacterium tuberculosis*, a pathogenic bacterium of the *Mycobacteriaceae* family and the causative agent of tuberculosis. The infection of macrophages with *M. tuberculosis*, in the presence or absence of TNF, causes apoptosis of the cells associated with a reduction in Fas expression (Fig. 1). Interestingly, the effect is observed with both an attenuated (H37Ra) or a virulent (H37Rv) strain of *M. tuberculosis*. In addition, apoptosis of infected macrophages diminished mycobacterial growth, which was not observed with a non-apoptotic cell death. Conversely, the interference of *M. tuberculosis* with FasL might also act on behalf of the bacterium as an escape mechanism to evade the effect of apoptosis (Oddo et al. 1998) (Fig. 1). The targeting of Fas as a strategy to avoid apoptosis has also been observed in *Brucella*, a Gram-negative facultative, intracellular coccobacillus, responsible for brucellosis, a worldwide zoonosis. Macrophages infected with *Brucella suis* are resistant to FasL or IFN-γ-induced apoptosis, which is interesting because the infection with this bacterium seems to protect host cells from some cytotoxic processes of the immune response (Fig. 1). Additionally, Bcl-2 also participates in the inhibition of apoptosis by *Brucella*, which is dependent on viable bacteria and not mediated by lipopolysaccharide (Gross et al. 2000). The interference with Fas/FasL is not restricted to bacteria. It has been shown that cells infected with *Toxoplasma gondii*, an obligate intracellular protozoan that has the ability to infect any nucleated cell, are resistant to multiple inducers of apoptosis (Fig. 1). These include Fas-dependent and Fas-independent CTL-mediated cytotoxicity, IL-2 deprivation, gamma irradiation, UV irradiation, and the calcium ionophore beauvericin. Also, for the inhibition to occur, live intracellular parasites and ongoing protein synthesis are necessary (Nash et al. 1998). Interestingly, in addition to the inhibition of apoptosis, it has been reported that this parasite can also induce it by increasing the expression of Fas/FasL both in infected and in uninfected cells in ocular toxoplasmosis. It has been shown that during the late stage of this pathology there is apoptosis and tissue damage in the retina that may be due to an increase in the expression of Fas and FasL. This increase seems to have a dual role; it is protective in the early stage since it helps to eliminate the relatively small number of tachyzoites and in the late phase contributes to the ocular damage (Hu et al. 1999).

As mentioned earlier, another receptor capable of inducing apoptosis through the extrinsic pathway is TNFR and it has been shown that both the receptor and its ligand (TNF-α) are important targets of intracellular pathogens for the modulation of apoptosis. TNF-α is a cytokine with a known dual effect. On the one hand, it induces pro-survival NF-κB signaling and, on the other hand, in cases of excessive stimulation, it promotes apoptosis (Aggarwal 2003). In the case of bacteria, the interference with TNF is exemplified by *Chlamydia trachomatis*, the most common sexually transmitted bacterium that can also cause blindness. The infection of cells with this bacterium blocks TNF-induced apoptosis (Fig. 1). Interestingly, the infection only affects some TNFR signaling pathways. Particularly the activation of NF-κB that initiates at the plasma membrane is unaffected, while the routes that occur subsequently to the internalization of TNFR and the formation of a cytosolic signaling complex are blocked. Thus, caspase 8 is not activated despite IAPs or cFLIP are inhibited by RNAi. But, the direct activation of caspase 8 by experimental over-expression of FADD abrogated the *C. trachomatis*-apoptosis inhibition effect (Waguia Kontchou et al. 2016). In the case of infection of human alveolar macrophages with *Mycobacterium tuberculosis*, apoptosis is induced by a TNF-α-dependent mechanism that represents a host defense strategy since it limits the growth of this intracellular pathogen (Fig. 1). Interestingly, apoptosis is differentially induced depending on the stage of infection and the virulence of *M. tuberculosis*. During the first stages of infection, the inhibition of host cell apoptosis is crucial for the pathogen to reproduce. Contrarily, in later phases of infection the induction of apoptosis is indeed induced through TNF-α (Keane et al. 1997) and may be favorable for the pathogen to exit the cell and infect neighbor cells. In relation to the virulence of *M. tuberculosis,* the virulent MTB strain H37Rv induces substantially less apoptosis than the attenuated strain H37Ra (Jamwal et al. 2013). This occurs through the release of soluble TNFR2 (sTNFR2) with formation of inactive TNF-alpha-TNFR2 complexes and is dependent on IL-10 (Balcewicz-Sablinska et al. 1998).

Another bacterium in which TNF plays a key role in promoting induced cell death is *Yersinia pseudotuberculosis*, a Gram-negative bacterium that causes Far East scarlet-like fever in humans. Interestingly, the infection of cells with *Y. pseudotuberculosis* inhibits TNF production, but apoptosis can still be induced by TNF produced by additional effector cells recruited to the site of infection or by surface TNF. TNFR1-induced apoptosis requires dynamin dependent endocytosis, which is presumably TRIF-independent and dependent on the formation of an intracellular DISC. Altogether these results suggest an unanticipated role of TNF and TNFR1in directing apoptosis of *Yersinia*-infected cells and promoting host protection against *Yersinia* infection (Peterson et al. 2016) (Fig. 1).

The use of TNFR/TNF-α as a target to modulate apoptosis is not limited to bacteria. It is known that the dimorphic fungus *Histoplasma capsulatum*, that mainly infects lung macrophages and reproduces as yeast, induces apoptosis through the extrinsic pathway through TNFR1/TNF-α (Fig. 1) and can be reduced by IL-0. The infection also activates caspases 3 and 1 that contribute to cell death, but only caspase 3 is involved in apoptosis. Interestingly, fungal burden is very important for the induction of apoptosis since at low infection quotients, it is barely detectable. Also, the blockade of apoptosis affects cytokine secretion but not fungal burden. These findings demonstrate that the time of infection interferes with the modulation of apoptosis. During the early stages of histoplasmosis, yeast invasion of lung macrophages induces apoptosis, triggered in part in an autocrine TNF-α–dependent manner, followed by release of IL-10 that likely prevents apoptosis of newly infected neighboring phagocytes (Deepe and Buesing 2012).

Besides the modulation of the most common inducers of the extrinsic pathway (Fas/FasL and TNF/TNFR) by pathogens, the interference with TRAIL has also been demonstrated. Such is the case of the Gram-negative bacterium, *Helicobacter pylori* that is associated with chronic gastritis, peptic ulcer, gastric carcinoma, and gastric mucosa-associated lymphoid tissue lymphomas. For these gastric pathologies, apoptosis induced by the infection of gastric epithelial cells with *H. pylori* is important. Besides triggering apoptosis, *H. pylori* enhances the assembly of TRAIL death-inducing signaling complex through downregulation of cellular FLICE-inhibitory protein and activates caspase 8. In addition, *H. pylori* infection promotes infiltration of T lymphocytes and inflammation that in turn increases apoptosis (Tsai and Hsu 2017).

#### Modulation of signaling pathways

In addition to the interference with the extracellular signals that activate apoptosis through the extrinsic pathway, pathogens have also developed mechanisms that target the signaling pathways initiated upon the ligation of the death receptors with their corresponding ligands. Different bacteria and parasites have been shown to interfere with various signaling pathways such as PI3K/AKT, the transcription factor NF-κB or the p53 protein. Some of these routes are activated by microbial pattern recognition or cytokine receptors and connected to pro-survival signals that restrain cell death pathways (Blander 2014). NF-κB is a transcription factor that is activated after the recognition of pathogen-associated molecular patterns (PAMPs) by the host pattern recognition receptors. It plays a key role in innate immunity through the control of the production of pro-inflammatory cytokines and cell death responses that can be pro- or anti-apoptotic depending on the context (Lin et al. 1999). For example, NF-κB activation induces the expression of pro-survival proteins such as TNFAIP8 (He and Ting 2002) and c-FLIP (Micheau et al. 2001).

**Fig. 2:**
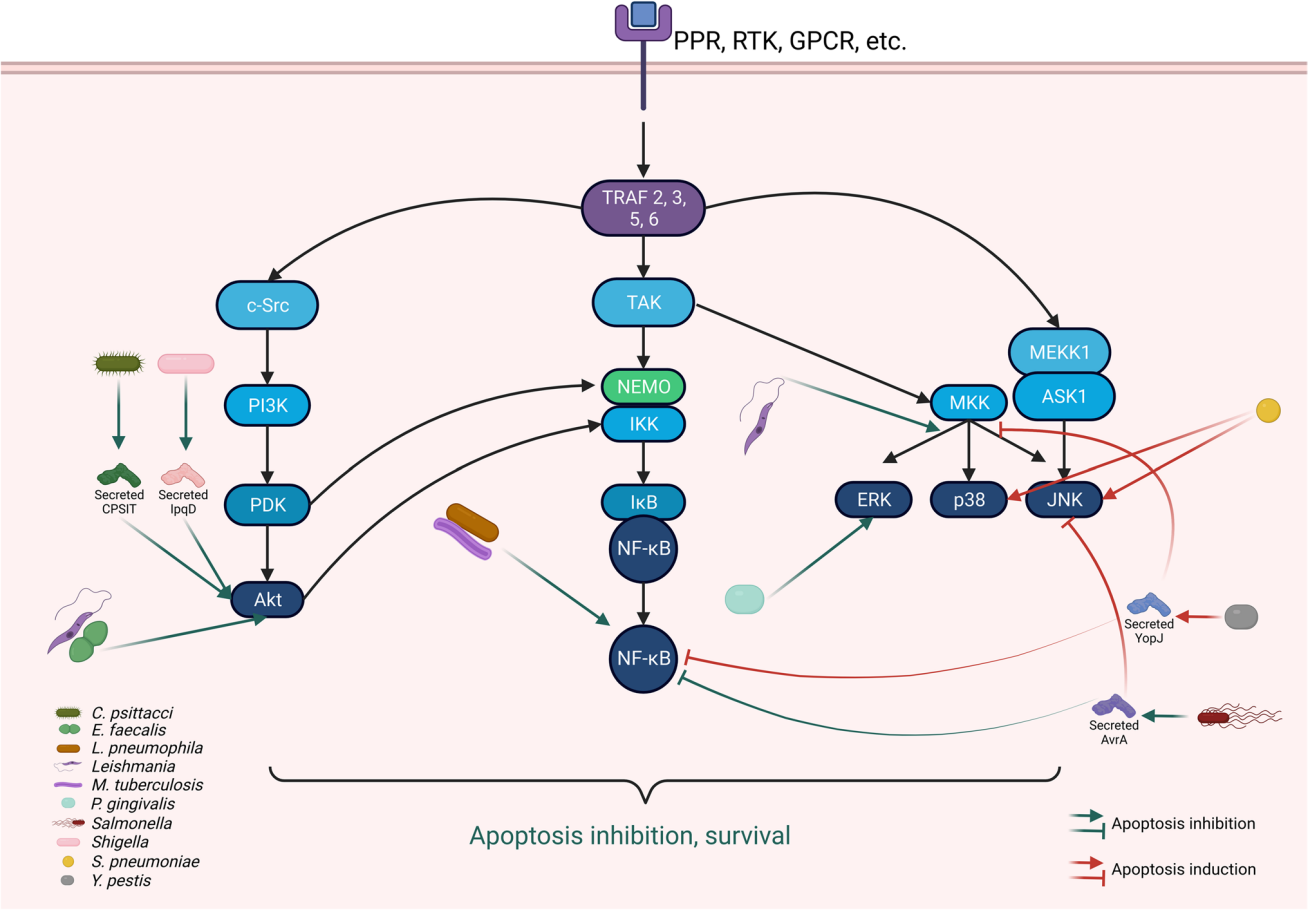
Modulation of signaling pathways. Apoptosis is a highly regulated process in which it is known that several signaling pathways participate, where the best studied are Akt, NF-κB, or MAPK. Generally, the activation of these signaling pathways conducts to the synthesis of proteins that inhibit apoptosis or to the inhibition of proteins that induce apoptosis. It has been observed that some pathogenic microorganisms can activate or inhibit these pathways in order to modulate host cell apoptosis. For example, *C*. *trachomatis*, *Leishmania*, and *Shigella* can activate Akt signaling pathway, while *Leishmania* and *S*. *pneumoniae* activate MAPK in order to inhibit host cell apoptosis. Interestingly, *Salmonella* can inhibit both NF-κB as well as JNK to delay the proapoptotic innate immune response. On the other hand, *L*. *pneumophila* and *M*. *tuberculosis* can activate NF-κB and in this way inhibit apoptosis. Contrarily, *Y*. *pestis* can inhibit both NF-κB and MAPK, which results in the induction of host cell apoptosis

Several bacteria have been shown to promote or interfere with apoptosis through the inhibition or activation of NF-κB by targeting it through type III or type IV secretion systems (Gao and Abu Kwaik 2000). In the case of activation, it is clearly exemplified by *Legionella pneumophila*, which is one of the main pathogens causing pneumonia and respiratory tract infections. Pathogenesis of *L*. *pneumophila* relies on a specialized translocation system known as the Dot/Icm type IV secretion system (TFSS). Interestingly, during the exponential phase of intracellular replication, *L*. *pneumophila* triggers a strong anti-apoptotic signaling cascade mediated, at least partially, by a sustained Dot/Icm-dependent and PAMP-independent NF-κB activation and up-regulation of anti-apoptotic genes, which renders the cells refractory to external potent apoptotic stimuli (Abu-Zant et al. 2007) (Fig. 2). Similarly, the virulence and pathogenicity of *M*. *tuberculosis* is associated with diverse secreted antigens such as the family of proteins ESAT-6 (esxA, esxT, and esxL). It has been shown that activation of NF-κB is involved in esxT-induced apoptosis (Kinhikar et al. 2010) (Fig. 2). Other studies have identified additional molecules, different from secreted antigens, such as the gene Rv2456c that may play a direct or indirect role in the inhibition of apoptosis in wild-type *M*. *tuberculosis* through activation of NF-κB. It has been shown that the lack of this protein probably fails to activate NF-κB, which enhances caspase-3 mediated apoptosis in infected cells (Jurcic Smith and Lee 2016). In the case of *Yersinia*, macrophages respond to infection with this pathogenic bacterium by activating MAPK- and NF-κB-signaling pathways. NF-κB promotes cell survival by up-regulating expression of several apoptosis inhibitor genes. To counteract this response, this bacterial genus delivers cytotoxic plasmid-encoded *Yersinia* outer proteins (Yops) to host cells to establish a successful infection. Yops achieve this through the blockade of different functions such as phagocytosis, cytokine responses, MAPK and NF-κB-signaling pathways and induction of apoptosis. Specifically, YopJ deactivates both pathways and suppresses TNF-α to promote rapid apoptosis important for bacterial dissemination from mucosal tissues (Monack et al. 1998; Zhang et al. 2005) (Fig. 2).

## Modulation through the interference with caspases

**Fig. 3:**
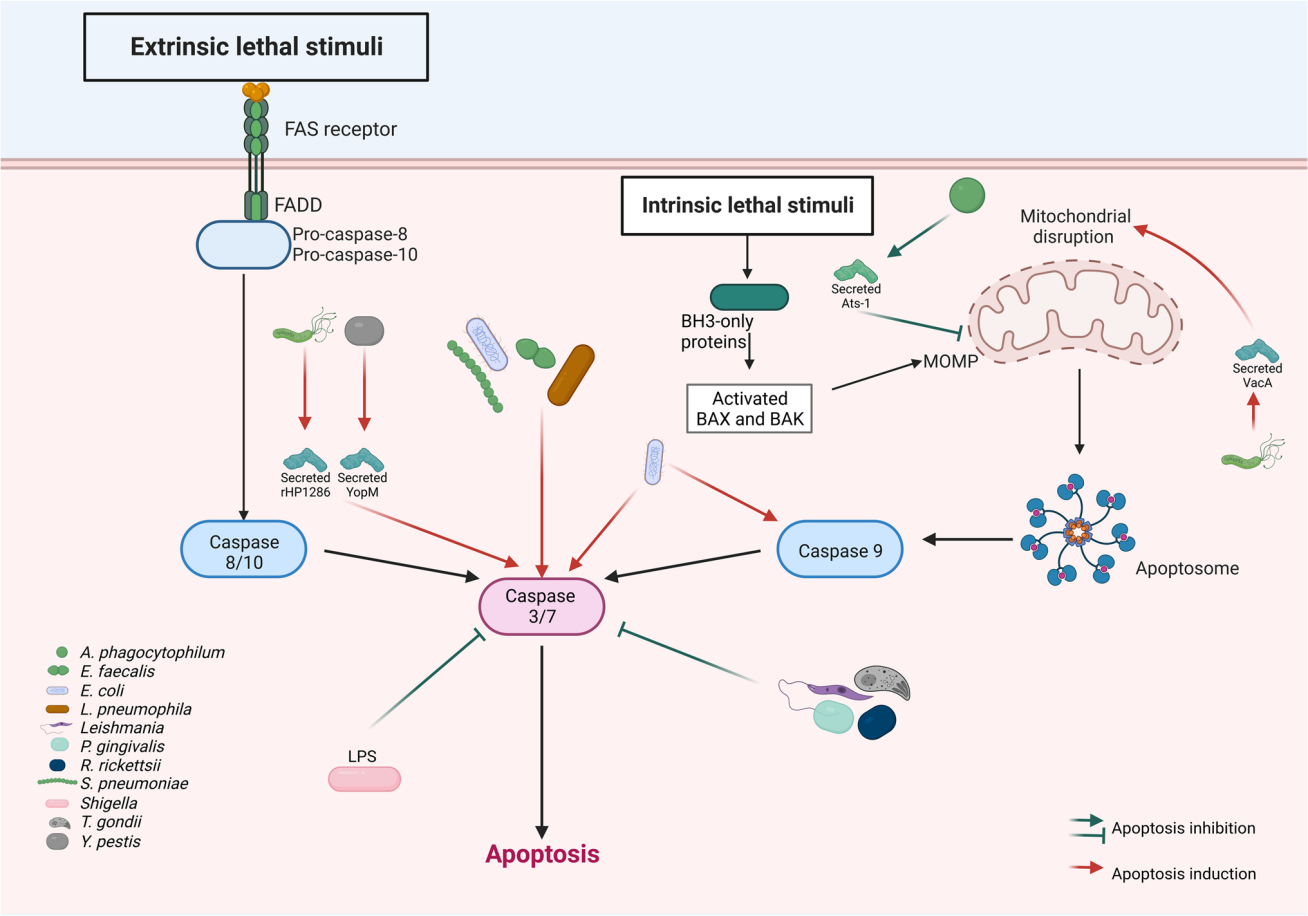
Modulation through the interference with caspases. The induction of apoptosis through both the extrinsic and the intrinsic pathway leads to the activation of the executioner caspases 3 and 7. Due to this, many intracellular pathogenic microorganisms modulate host cell apoptosis through the interference with caspases. For example, it has been observed that *Leishmania* and *P*. *gingivalis* inhibit caspase 3. On the other hand, it has been observed that *E*. *coli*, *L*. *pneumophila*, and *Y*. *pestis* inhibit caspase 3, and *E*. *coli* can also inhibit caspase 9

Caspases are crucial participants in the process of apoptosis responsible for activating the dismantling of the cell. Thus, they are important targets for the modulation of apoptosis by intracellular pathogens. Due to the preponderant role of caspase 3, where the different activation pathways converge, it is not surprising that it is one of the main targets of intracellular pathogens to modulate apoptosis, in some instances to induce it and in others to inhibit it. The interference with caspases as a strategy to modulate apoptosis has been demonstrated during infection with several bacteria. As examples, phagocytosis of RAW264.7 cells with *Escherichia coli* laboratory strain K12 DH5 alpha induces apoptosis in approximately 70% of the cells after 24-h post-exposure and the inhibition of caspases blocked the induction of apoptosis. Interestingly, the caspases that were activated were 3 and 9, but not 8, which suggests that apoptosis was induced through the intrinsic pathway (Häcker et al. 2002) (Fig. 3). Also, YopM from *Yersinia pestis*, the causative agent of bubonic plague, induces a pathogenic effect in the liver and spleen by promoting caspase-3-dependent apoptosis of polymorphonuclear leukocytes (PMN’s) and bacterial growth. YopM apoptotic effect acts directly or indirectly with or downstream of activated caspase-3 (Fig. 3). This apoptotic effect may represent a pathogenic mechanism since PMNs limit *Y. pestis* growth in the liver. It seems that YopM can move the balance toward caspase-3 activation and death of PMNs (Ye et al. 2014). Another bacterium, *Porphyromonas gingivalis*, a Gram-negative anaerobic bacterium that is the major etiologic agent in chronic periodontitis, regulates apoptosis of human monocytic THP-1 cells induced under growth factor deprivation. The regulation of apoptosis is carried out by certain bacterial cell components and is associated with chronic inflammation in this disease. In particular, bacterial fimbria, an important cell structure involved in adherence to host cells, inhibits the serum withdrawal-induced cleavage of the caspase-3 proform (Fig. 3). The inhibitory action of the fimbriae was completely neutralized by anti-fimbrial antibody and inhibited by treatment with Ac-DEVD-CHO, an inhibitor of caspase-3. Bacterial fimbria also cleaved poly (ADP-ribose) polymerase (PARP), which is a nuclear protein that plays various critical roles in different cellular processes including DNA repair, genomic stability, cell cycle control, apoptosis, and necrosis (Hassa et al. 2006) and is a substrate of caspase 3/7-mediated cleavage during apoptosis (Ozaki and Hanazawa 2001).

Other bacteria where caspase 3 is involved in the modulation of apoptosis are *L*. *pneumophila Rickettsia rickettsia* and *H. pylori* (Fig. 3). The activation of caspase 3 by *L*. *pneumophila* is very interesting and closely related to the ratio and temporality of infection. It has been shown that the infection of macrophages with low loads of *L*. *pneumophila* causes an early and strong activation of caspase 3 through the Dot/Icm secretion system independent of the classical extrinsic and intrinsic signaling pathways of apoptosis (Abu-Zant et al. 2005). Its function is more related to the evasion of the endosomal-lysosomal pathway than to the activation of apoptosis. Contrarily, higher infection loads trigger apoptosis through the intrinsic pathway that halts intracellular replication (Neumeister et al. 2002; Zink et al. 2002; Abu-Zant et al. 2005). Thus, this delay of apoptosis in *L*. *pneumophila*-infected macrophages, despite caspase 3 activation, suggests that *L*. *pneumophila* may modulate anti-apoptotic signaling to permit intracellular replication for a longer period (Fischer et al. 2006). On the other hand, *Rickettsia rickettsii* is a tick-borne obligate intracellular bacterium that causes Rocky Mountain spotted fever, a life-threatening illness. The modulation of apoptosis by this bacterium was analyzed in infected proteome cells of the tick embryonic cell line BME26 where it was shown that both caspase-3 activity and phosphatidylserine exposition were lower in infected than in noninfected cells. The activation of caspase-3 exerted a detrimental effect on rickettsial proliferation and its inhibition increased bacterial growth. Also, the expression of other negative regulators of apoptosis changed during the course of infection (Martins et al. 2020). In the case of *H*. *pylori*, the persistent infection of macrophages diminishes the number of cells through the induction of apoptosis, which protects bacteria from the microbicidal activities of macrophages. The factor responsible for the induction of apoptosis of macrophages in a dose- and time-dependent manner was the protein rHP1286. This protein caused significantly higher caspase 3 activity compared to macrophages infected with a mutant strain of *H. pylori* lacking HP1286 protein (Fig. 3). Interestingly, macrophage apoptosis was TNF-independent since it was not inhibited with neutralizing antibodies against TNF (Asim et al. 2010)

Another bacterium, *Enterococcus faecalis*, belongs to the commensal flora, nevertheless, possesses virulence factors, that make it an opportunistic pathogen related to hospital-acquired wound, urinary tract, and bloodstream infections (Gjødsbøl et al. 2006; Kayaoglu and Ørstavik 2004; Wisplinghoff et al. 2004). *E. faecalis* infection prevents apoptosis of macrophages through the activation of caspase 3 that was observed at 6- and 12-h post-infection (Fig. 3). The expression of other proteins, markers of pyroptosis (NLRP3, gasdermin D [GSDMD] and caspase-1) and necroptosis (mixed lineage kinase domain-like protein [MLKL] and receptor interacting protein kinase 3 [RIPK3]) were also observed (Chi et al. 2021).

Also, the cytosolic Gram-negative bacterium that colonizes the intestinal epithelium *Shigella flexneri* halts apoptosis through the inhibition of effector caspase activity. The inhibition of caspases is carried out by lipopolysaccharide (LPS) that directly binds caspases through its O-antigen (O Ag) moiety. Bacterial strains that lacked the O Ag or failed to replicate within the cytosol were incapable of blocking apoptosis and exhibited reduced virulence in a murine model of bacterial infection (Günther et al. 2020) (Fig. 3).

In addition to bacteria, several intracellular parasites modulate apoptosis through caspases. Such is the case of *Toxoplasma* that blocks both the activity and activation of caspase 3 and upstream caspase (8 and 9) in infected cells (Goebel et al. 2001; Payne et al. 2003) (Fig. 3).

Also, the infection with *T*. *gondii* downregulates the expression of PARP-1 that coincides with a complete resistance of infected cells against the induction of apoptosis (Gais et al. 2008).

Another intracellular parasite, *Leishmania*, transmitted through the bite of phlebotomine sandflies and the causative agent of a group of diseases called leishmaniasis, infects different cells such as macrophages, dendritic cells, and neutrophils. Upon inoculation into a mammalian host, *Leishmania* parasites must adapt to this novel environment and take advantage of its components and cells through different approaches, one being a delay in apoptosis induction in neutrophils, macrophages and dendritic cells (Aga et al. 2002; Moore and Matlashewski 1994; Valdés-Reyes et al. 2009). This implies that the parasite induces changes in the cellular signaling machinery early during the infectious process to avoid activation of the apoptotic program. In the case of neutrophils, it has been shown that the coincubation of PMN with *L. major* promastigotes in vitro led to an inhibition of their spontaneous apoptosis, which was demonstrated by morphology of apoptotic PMN, annexin V binding, and DNA fragmentation. The survival of PMN increased after 42 h of coincubation with *Leishmania* through a mechanism involving the inhibition of caspase-3 activation (Aga et al. 2002) (Fig. 3).

Also, our group has demonstrated that apoptosis of monocyte-derived dendritic cells (moDC) induced by treatment with camptothecin was downregulated by coincubation with *L. mexicana*, as detected by annexin V binding to phosphatidylserine, morphological analysis of cell nuclei, and DNA fragmentation. The observed antiapoptotic effect was found to be associated with a significant reduction of caspase-3 activity in moDC (Valdés-Reyes et al. 2009) (Fig. 3). The capacity of *Leishmania* to delay apoptosis induction in the infected cells may have implications for *Leishmania* pathogenesis by favoring the invasion of its host and its persistence in the infected cells.

### Interference with MAPK

Mitogen-activated protein kinases (MAPK) participate in crucial molecular events in the cell such as growth, proliferation, production of inflammatory cytokines, and apoptotic cell death (Gao et al. 2012; Solano-Gálvez et al. 2018). Due to their participation in apoptosis, several intracellular microorganisms interfere with their activation in order to modulate apoptosis. Among MAPK, c-Jun N-terminal kinase (JNK) plays a pivotal role in the induction of apoptosis, thus targeting it to dampen apoptosis may be a conserved strategy for intracellular pathogens to modulate the induction of this type of cell death. Starting with bacteria, a clear example of the interference with MAPK is found in *Salmonella typhimurium*. Salmonellae are very interesting bacterial pathogens that have an enormous ability to subvert innate immune and apoptotic signaling pathways for which have evolved sophisticated strategies that include the secretion of effector proteins into mammalian cells. One of these secreted effector proteins is AvrA that has acetyltransferase activity toward MAPK. In experimental models of infection with *S. typhimurium* of both transgenic *Drosophila* and mice, AvrA strongly inhibits JNK and NF-κB signaling pathways. Interestingly, during the natural course of infection of mammalian hosts with *Salmonella*, it elicits transient inflammation, but do not destroy epithelial cells. This occurs, in part, because in the intestinal mucosa of mice AvrA decreases the pro-apoptotic innate immune response to *Salmonella* (Wu et al. 2012) (Fig. 2). Another example of deactivation of MAPK is observed in *Yersiniae*. The infection of macrophages with pathogenic *Yersinia* species activates MAPK (p38 and JNK) and NF-κB that promote the expression of survival genes. In response, bacteria secrete YopJ that deactivates MAPK-signaling pathways and induces apoptosis (Orth 2002; Zhang et al. 2005) (Fig. 2). Particularly, YopJ strongly inhibits the expression of many genes, especially those encoding cytokines and six apoptosis-related genes such as Phlda1 and Plagl1, that encode apoptosis promoting factors (Hossain et al. 2003; Spengler et al. 1997).

As already mentioned, the *H. pylori* HP1286 protein induces apoptosis of macrophages. The induction of apoptosis occurs, in part, through the modulation JNK and extracellular signal-regulated kinase (ERK) as demonstrated with the pre-treatment of macrophages with U0126, an inhibitor of the ERK, that significantly reduced nuclear translocation of ERK and phosphorylation of c-Fos in rHP1286-treated macrophages. It is suggested that the ability of HP1286 to induce macrophage apoptosis could possibly help in the bacterial escape from the activated macrophages and persistence in the stomach (Tavares and Pathak 2017) (Fig. 2). Another example of interference of MAPK by bacteria is observed in *Streptococcus pneumoniae*, which, as already mentioned, provokes apoptosis of endothelial cells. It has been shown that p38 and JNK were activated in pneumococci-infected cells and inhibitors of both kinases strongly reduced pneumococci-induced caspase activation and apoptosis. Hence, kinase- and caspase-dependence of pneumococci-induced endothelial apoptosis may represent novel therapeutic approaches to pneumococci-related disease (N’Guessan et al. 2005) (Fig. 2).

As mentioned earlier, *P. gingivalis* fimbriae inhibits THP-1 cells apoptosis induced under growth factor deprivation. In addition to caspase 3, other mechanism involved in the inhibition of apoptosis is the activation of (ERK) (Fig. 2) and expression of cyclin-dependent kinase inhibitor p21 Cip/WAF1 (p21) in the cells. The stimulatory effect of the fimbriae on the expression of the p21 protein was inhibited by treatment with PD98059, a specific inhibitor of ERK, which along an antisense p21 oligonucleotide blocked the fimbrial inhibition of apoptosis (Ozaki and Hanazawa 2001).

As with other interferences of signaling pathways, targeting of MAPK is not restricted to bacteria. One intracellular parasite in which the participation of MAPK in the inhibition of apoptosis has been widely documented is *Leishmania*. We have shown that *L. mexicana* amastigotes and promastigotes inhibit monocyte-derived dendritic cells apoptosis. One of the strategies employed by the parasite is the deactivation of JNK and p38 that diminished significantly DNA fragmentation in moDC stimulated with camptothecin (Rodríguez-González et al. 2016; Vázquez-López et al. 2015) (Fig. 2). Additionally, it has been demonstrated that the inhibition of pro-apoptotic signaling through p38 exerted by *Leishmania* can also occur with certain soluble structural components of the protozoan, such as the surface protein gp63 (Hallé et al. 2009).

### Modulation of PI3K/Akt

The PI3K/Akt signaling pathway is involved in proliferation, differentiation, and downregulation of apoptosis making it a decisive participant in cellular survival. Its involvement in apoptosis makes it another important target for pathogens to modulate this type of cell death. One interesting example of modulation through this pathway is observed in *Shigella.* This bacterium possesses an effector molecule, IpgD, with phosphoinositide phosphatase activity that transforms phosphatidylinositol 4,5-biphosphate (PIP2) to phosphatidylinositol 5-phosphate (PIP5) (Tran Van Nhieu et al. 2022). PIP5 promotes the activation of the epidermal growth factor receptor (EGFR) that sustains the pro-survival pathway, PI3K/Akt, and prevents the induction of apoptosis. In particular, it has been shown that *S. flexneri* injects IpgD, PIP5 phosphorylates host cell Akt, which depends on the activation of a class IA PI 3-kinase via tyrosine phosphorylations and colocalizes with the active form of this kinase during the initial steps of infection. In this way, IpgD-mediated pathways protect cells from apoptosis through direct and transcriptional control of antiapoptotic processes. This mechanism is particularly important to regulate the survival of infected cells in order to maintain efficient bacterial colonization (Pendaries et al. 2006) (Fig. 2). Another example is observed with *Chlamydia psittacci* that can cause severe pneumonia and bacteremia in humans. *Chlamydia spp* possess membrane inclusion proteins that play an important role in the interaction with the host. One of these proteins, CPSIT_0556, has been shown to inhibit neutrophils apoptosis through the PI3K/Akt and NF-κB signaling pathways (He et al. 2021) (Fig. 2). Also, it has been shown that *E. faecalis* infection may block apoptosis of macrophages by activating PI3K signals (Zou and Shankar 2014) (Fig. 2).

In regard to the modulation of PI3K/Akt pathway by a parasite, our group has shown that the infection of monocyte-derived dendritic cells with *L. mexicana* activates this pathway as a strategy to inhibit apoptosis of the cells (Vázquez-López et al. 2015) (Fig. 2). This effect is not particular of dendritic cells or *L*. *mexicana* since it has been demonstrated that the infection of bone marrow macrophages with *L*. *major* or *L*. *pifanoi* also activates PI3K/Akt (Ruhland et al. 2007). We recently demonstrated the involvement of Akt in the inhibition of apoptosis of dendritic cells by *L*. *mexicana*. We showed that the inhibition of Akt reverts the apoptosis protective effect exerted by *L*. *mexicana* on moDC reflected by a reduction in MOMP, caspase 3 activation, and upregulation of Bcl-xL (Rodríguez-González et al. 2022). Also, *L. donovani* inhibits host apoptosis by targeting Akt that modulates its downstream transcription factors, β-catenin and FOXO-1. The infection of RAW264.7 and bone marrow-derived macrophages (BMDM) with *L. donovani* activates Akt. Phospho-Akt in turn inactivates GSK-3β that could no longer sequester cytosolic β-catenin, an anti-apoptotic transcriptional regulator, and inhibits activation of FOXO-1, a pro-apoptotic transcriptional regulator. The activation of Akt exerted by *L. donovani* to regulate the GSK-3β/β-catenin/FOXO-1 axis ensures intra-macrophage survival through inhibition of both host cell apoptosis and immune response (Gupta et al. 2016).

### TLR signaling

The activation of TLRs not only plays an important role in innate immunity, but it is also linked to the induction of autophagy, apoptosis, or pyroptosis in different cells. Thus, it is not strange that some microorganisms target TLR’s signaling pathways to modulate apoptosis. One example is the enteropathogenic bacterium *Yersinia enterocolitica* that deactivates TLR-induced signaling pathways, which triggers apoptosis in macrophages. *Yersinia*-induced apoptosis of human macrophages involves caspase-dependent cleavage of the TLR adapter protein MyD88, which is also cleaved when apoptosis is mediated by overexpression of the Toll–IL-1R domain–containing adapter inducing IFN-γ in epithelial cells (Novikova et al. 2014). In addition to Yops, apoptosis can be triggered by TLR4/TRIF, although significant cell death may occur in *Yersinia*-infected cells even in the absence of TLR4/TRIF-dependent signals (Zhang and Bliska 2003; Haase et al. 2003; Ruckdeschel et al. 2004, which requires dynamin dependent endocytosis.

## Modulation of the intrinsic pathway by intracellular pathogens

As already stated, the main trigger of the intrinsic pathway of apoptosis is cellular stress manifested in different facets such as DNA damage and oxidative stress. Due to the ambiance where intracellular pathogens develop, they are continuously exposed to cellular stress and the induction of apoptosis. Thus, it is not astounding that many of them inhibit apoptosis by intervening with this pathway. Interestingly, the generation of cellular stress starts with the infection itself that induces both damage to the DNA and oxidative stress (Pitangui et al. 2016; Rodríguez-González et al. 2018) and subsequently apoptosis. Possibly, this is one of the reasons why intracellular pathogens are capable of inhibiting this pathway. A clear example of damage of host DNA is observed in the infection of *Shigella* of epithelial cells followed by the activation of apoptosis induced by p53. Interestingly, *Shigella* prevents the activation of apoptosis by the secretion of the VirA effector, which degrades it to calpastatin, a calpain inhibitor. Active calpain degrades it to p53 and prevents the activation of apoptosis through this pathway (Bergounioux et al. 2012). In addition to host DNA damage by the infection with intracellular pathogens, mitochondria are another important target. Mitochondria are complex organelles that participate in a plethora of functions, ranging from ATP production to immune responses against viruses and bacteria. Thus, being an organelle that integrates so many functions makes mitochondria a very attractive target to manipulate for intracellular pathogens. A number of pathogenic bacteria target mitochondria to modulate the host’s apoptotic machinery as has been shown with (e.g., *Listeria monocytogenes* and *Vibrio cholera*) and viruses (e.g., *Hepatitis* C and Epstein-Barr) (Escoll et al. 2016; Fielden et al. 2017; Khan et al. 2015). As mentioned before, the stability of the mitochondria is crucial for the developing of apoptosis. If altered, it can promote (Scorrano 2013) or protect from apoptosis (Szabadkai et al. 2004). In addition of inducing apoptosis, the alteration of mitochondrial morphology can also generate a metabolic slowdown of the host cell or interfere with the immune signaling as has been observed in different bacteria such as *Brucella* (Lobet et al. 2018), *Helicobacter pylori* (Jain et al. 2011) or *Lepstospira* (Hu et al. 2017). In the case of *Brucella*, the infection of cells causes physical contacts between *Brucella-*containing vacuoles and mitochondria. In particular, *B. abortus* and *B. melitensis* induce a drastic mitochondrial fragmentation at 48-h post-infection in different myeloid and non-myeloid cells. This fragmentation is dynamin-related protein-1 (DRP1)-independent and might be caused by a deficit of mitochondrial fusion. However, fragmentation of the mitochondria does not affect the sensitivity of *Brucella*-infected cells to TNF-α-induced apoptosis, nor does it impair replication of the bacteria in the host cells (Lobet et al. 2018). On the other hand, infection with *H. pylori* disrupts the morphological dynamics of mitochondria as a mechanism to induce host cell death. The disruption occurs through the vacuolating cytotoxin A (VacA), a mitochondrial acting exotoxin, that induces mitochondrial network fragmentation through the mitochondrial recruitment and activation of dynamin-related protein 1 (Drp1). At the same time, DRp1 is a critical regulator of mitochondrial fission within cells. Inhibition of Drp1-induced mitochondrial fission within VacA-intoxicated cells inhibited the activation of the proapoptotic Bcl-2–associated X (Bax) protein, permeabilization of the mitochondrial outer membrane, and cell death. This mechanism represents a novel strategy by which a pathogenic microbe engages the host’s apoptotic machinery (Jain et al. 2011).

Another example of induction of apoptosis by mitochondrial damage is *Leptospira.* It has been shown that in leptospire-infected macrophages mitochondrial apoptosis-inducing factor (AIF) and endonuclease G (EndoG) are released from the mitochondria and translocated to the nucleus leading to nuclear DNA fragmentation, which is preceded by the activation of caspase-8 by the BH3-interacting domain death agonist (Bid). Also, infection triggers high reactive oxygen species (ROS) that dephosphorylates Akt, which also activates Bid (Hu et al. 2017).

### Secretion of bacterial proteins that inhibit the intrinsic pathway

**Fig. 4:**
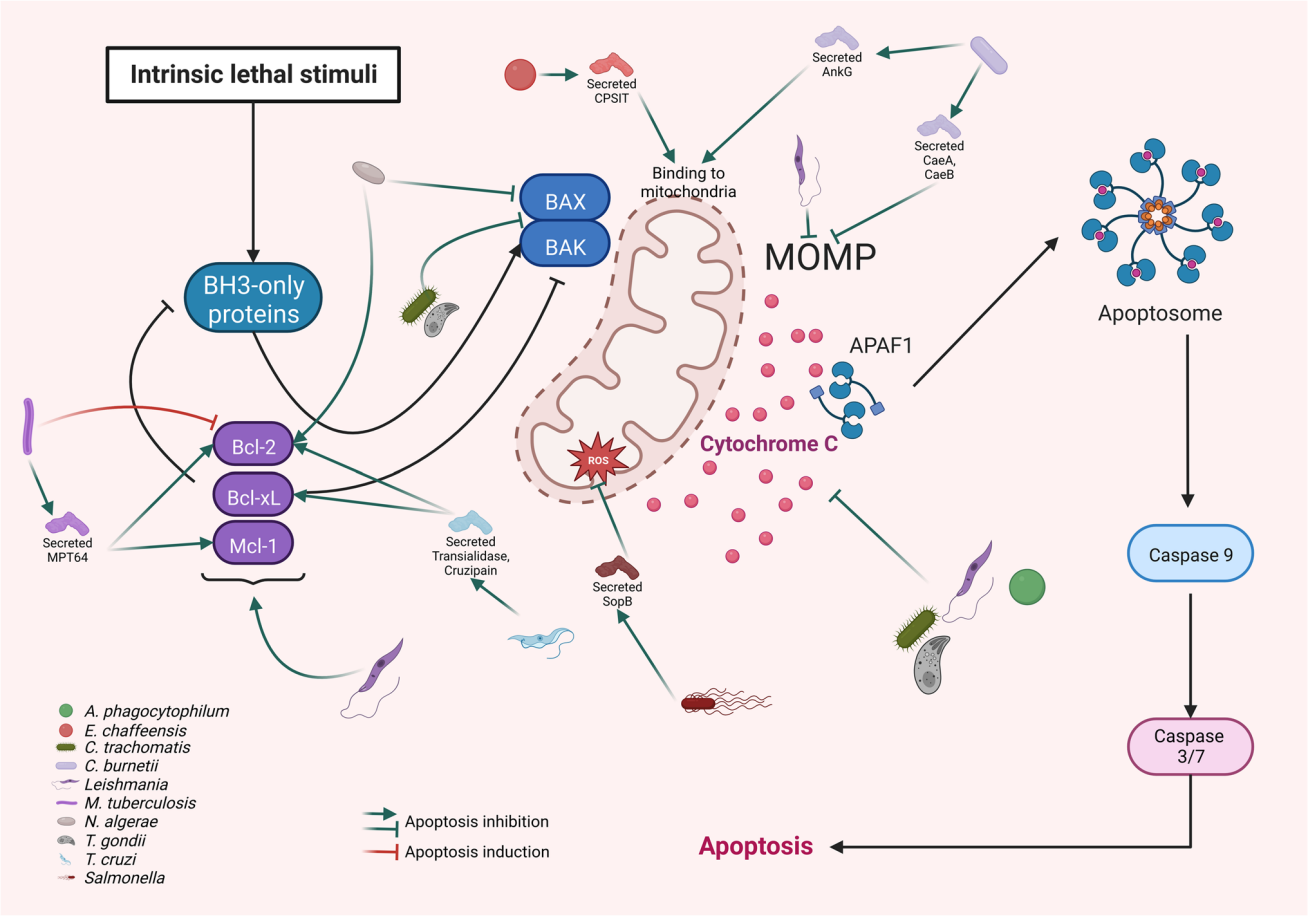
Modulation of proteins that regulate MOMP by intracellular pathogens. The induction of apoptosis through the intrinsic pathway leads to the loss of the mitochondrial outer membrane permeability (MOMP), which permits the release of lethal molecules, as for example cytochrome c, that along with APAF1 form the apoptosome, which permits the autoactivation of caspase 9 and finally the activation of caspases 3 and 7. Interestingly, the activation of the intrinsic pathway can occur through the extrinsic pathway thanks to a positive feedback loop that depends on the formation of tBID from BID. MOMP is a mechanism characterized by a fine regulation carried out by different proteins, mainly by proteins that belong to the Bcl-2 family that participate in regulatory processes such as post-translational modifications and regulation of the expression of different genes, as those of the Bcl-2 family, that finally will decide the fate of the cell. Due to all this, intracellular pathogens have developed different strategies to modulate the MOMP, the molecules released from the mitochondria, and proteins from the Bcl-2 family. For example, it has been observed that *Leishmania*, *M*. *tuberculosis*, *N*. *algerae*, and *T*. *cruzi* induce the overexpression of the antiapoptotic proteins Bcl-2, Bcl-xL, and Mcl-1 and in this way inhibit host cell apoptosis, although it has also been observed that *M*. *tuberculosis* can induce apoptosis through the proapoptotic protein Bim. On the other hand, it has been shown that both *C*. *burnetii* and *Leishmania* can inhibit MOMP. In addition, it is known that *A*. *phagocytophilum*, *C*. *trachomatis*, *Leishmania*, *T*. *gondii*, and *Salmonella* inhibit the release of cytochrome c

In addition to the modulation of the intrinsic pathway of apoptosis through the alteration of mitochondria integrity, this pathway of apoptosis can also be disturbed by proteins secreted by several bacteria. One example is *Anaplasma phagocytophilum*, the causative agent of human granulocytic anaplasmosis, that infects human neutrophils and inhibits mitochondria-mediated apoptosis through an effector named Anaplasma translocated substrate 1 (Ats-1). Ats-1 is abundantly expressed by *A. phagocytophilum*, is translocated either to the internal mitochondrial membrane or the mitochondrial matrix, prevents the release of cytochrome C, PARP cleavage, and in this way inhibits etoposide-induced host cell apoptosis (Niu et al. 2010) (Fig. 4). *Ehrlichia chaffeensis* is another bacterium that secretes an effector protein that perturbs mitochrondrial integrity. It causes human monocytic ehrlichiosis transmitted by a tick infected with *Ehrlichia* organisms that infect monocytes/macrophages. The effector protein that has been described is ECH0825 and shown to be translocated from the bacterium into the host-cell cytoplasm, localized to mitochondria and inhibits human Bax-induced apoptosis. Also, in *E. chaffeensis*-infected cells and ECH0825-transfected cells, there is an important increase (over 9-fold) in mitochondrial manganese superoxide dismutase (MnSOD) and contrarily the amount of ROS was significantly lower. The upregulation of MnSOD by ECH0825 prevents ROS-induced cellular damage and apoptosis to allow intracellular infection (Liu et al. 2012) (Fig. 4).

In the case of *Coxiella burnetii*, an intracellular pathogen of mammalian hosts that causes an acute disease, Q-fever, the anti-apoptotic activity has been shown to depend on a functional type IV secretion system (T4SS), which is believed to be important for the establishment of a persistent infection. Two proteins belonging to this secretion system, *C. burnetii* anti-apoptotic effectors (CaeA and CaeB) have been identified that participate in the inhibition of natural or induced apoptosis. CaeB inhibits staurosporine-induced apoptosis through the efficient inhibition of MOMP, while the anti-apoptotic activity of CaeA is weaker (Klingenbeck et al. 2013). Another protein described in *Coxiella* is AnkG that inhibits pathogen-induced apoptosis, possibly by binding to the host cell mitochondrial protein p32 (gC1qR). It is crucial for the migration of AnkG to the nucleus along with Importin-α1 that is essential for migration and nuclear localization of AnkG and its anti-apoptotic activity. Interestingly CaeB does not bind to p32 (Schäfer et al. 2017) (Fig. 4). Other microbial pathogens enter host cells by injecting effector proteins of the Type III secretion system (T3SS), which facilitate pathogen translocation across the host cell membrane, modulate a variety of host innate immune responses, and facilitate bacterial replication and systemic infection. Such is the case of *Salmonella enterica* serovar typhimurium (*S. typhimurium*), a clinically important pathogen that causes food poisoning and gastroenteritis. The effector protein SopB is encoded in the *Salmonella* pathogenicity islands (SPI)-1 T3SS and protects host epithelial cells from infection-induced apoptosis. SopB attenuates the production of ROS by preventing the mitochondrial recruitment which leads to increased Bax/Bcl-2 ratio, Bax recruitment to mitochondrial membrane, and release of cytochrome c into the cytoplasm (Ruan et al. 2016) (Fig. 4).

The inhibition of apoptosis initiated by the intrinsic pathway not only is a target for pathogenic intracellular bacteria, but also for intracellular parasites. Such is the case of *Toxoplasma gondii* that targets activation of pro-apoptotic Bax and Bak to inhibit the apoptogenic function of mitochondria to increase host cell viability. The impairment of the activation of Bax and Bak, was not mediated by the upregulation of anti-apoptotic Bcl-2-like proteins following infection. Also, in infected epithelial cells with *T. gondii* BimS-triggered release of cytochrome c from host-cell mitochondria into the cytosol is abrogated in a dose-dependent manner (Hippe et al. 2009) (Fig. 4).

In infected cells with another intracellular obligate parasite, *Leishmania donovani*, it has been shown that the MOMP is inhibited (Gupta et al. 2016) and in the case of the infection with *L*. *major* it prevents the release of cytochrome C (Sarkar et al. 2013) (Fig. 4). The studies performed with protozoa and fungi differ significantly from what has been published regarding bacteria and the role of their secreted proteins in the inhibition of apoptosis, because there is not much information about the possible molecules that modulate host cell apoptosis.

### Modulation of proteins that regulate MOMP by intracellular pathogens

It has been already pointed out that MOMP is a no return way during the activation of apoptosis and thus its fine tuning is transcendental for the correct development of the process. The accuracy of MOMP is achieved thanks to the participation of proteins of the Bcl-2 family. As expected, members of this family are prey for intracellular microorganisms to induce or inhibit apoptosis. A good example of this modulation is found in members of the *Mycobacteriaceae* family where the infection of macrophages with *Mycobacterium bovis* or the incubation with heat-inactivated bacilli of *M*. *tuberculosis* trigger apoptosis in macrophages that results in death of the microorganism. The apoptotic-inducing effect of *Mycobacterium* was also demonstrated in vivo where significantly more apoptosis was observed in five tuberculosis patients compared to normal controls. The protein implicated in the induction of apoptosis was the antiapoptotic protein Bcl-2 as shown by the downregulation of Bcl-2 RNA or protein in peripheral blood monocytes (PBM) following infection with *M. bovis* BCG or induction with heat-killed *M. tuberculosis* H37Ra (Klingler et al. 1997) (Fig. 4).

Moreover, the infection of cells with *M*. *tuberculosis* can also induce host cell apoptosis through the proapoptotic protein Bim (Aguiló et al. 2014). Contrarily, the infection with *M*. *tuberculosis* can also induce the overexpression of Bcl-2 and Mcl-1 to inhibit host cell apoptosis. In particular, the overexpression of Bcl-2 has been associated with the secretion of MPT64 protein by *M*. *tuberculosis* (Sly et al. 2003; Wang et al. 2014). Once again, we find a situation where the same microorganism can induce or inhibit apoptosis. In this particular case through the modulation of both anti-apoptotic and pro-apoptotic proteins that result crucial for the success of the bacillus during the establishment of infection.

Another bacterium, *Legionella pneumophila* induces host cell apoptosis through the intrinsic pathway by targeting Bcl-xL, which has been demonstrated with the usage of a Bcl-xL inhibitor. Interestingly, this inhibition reduces the reproductive capacity of *Legionella* (Speir et al. 2016). *Leptospira* is another example of modulation of apoptosis through intervention with proteins of the Bcl-2 family. As already mentioned, the infection of host cells with this bacterium causes mitochondrial damage and the subsequent release and translocation of AIF and/or EndoG, which is preceded by the activation of the pro-apoptotic protein Bid by caspase 8. Additionally, the infection triggers the production of ROS that dephosphorylate Akt, which in turn activates Bid (Hu et al. 2017). Also, *Chlamydia trachomatis* strongly inhibits mitochondrial apoptosis of its host cell to secure its survival in human cells through the activation of Bak and Bax. Specifically, the infection of cells with *C. trachomatis* diminished the oligomerization and association with the outer mitochondrial membrane of Bax and the conformational changes of Bak. Interestingly, the protective effect of the infection was reduced in cells lacking the Bax/Bak-regulator VDAC2. Also, mitochondria isolated from *Chlamydia*-infected cells were protected against Bim and tBid, but this protection was lost upon protease digestion. OmpA, a porin of the outer membrane of *C. trachomatis*, was also effective in the inhibition of apoptosis, replicating the effect of the infection (Waguia Kontchou et al. 2022) (Fig. 4).

The modulation of proteins that regulate MOMP is not restricted to bacteria as it has also been observed in fungi and parasites. A clear example of this regulation by a fungus is found in the parasitic fungus *Nosema algerae* that infects human fibroblasts and causes the overexpression of Bcl-2 and a decrease in the expression of Bax, which finally results in the inhibition of apoptosis (Scanlon et al. 1999) (Fig. 4). Regarding the modulation of MOMP by a parasite, *Trypanosoma cruzi* is a very interesting example. This intracellular protozoan parasite infects several cells types, preponderantly macrophages and cardiomyocytes, and is the causative agent of Chagas disease. It secretes a transialidase that induces an overexpression of the anti-apoptotic protein Bcl-2 and thus inhibits host cell apoptosis, possibly through the activation of PI3K/Akt signaling pathway (Chuenkova and Pereira 2000). In addition to the secretion of the transialidase, *T*. *cruzi* secretes another enzyme with protease activity called cruzipain, which induces the overexpression both of Bcl-2, through ERK1/2 signaling pathway, and of Bcl-xL. Although for Bcl-xL, it has not been possible to implicate neither ERK1/2 pathway nor PI3K (del Pilar Aoki et al. 2006) (Fig. 4). In the case of another intracellular parasite, *Leishmania major,* it has been shown that the infection of cells causes the overexpression of the antiapoptotic proteins Bcl-2 y Bfl-1, possibly through the ERK1/2 signaling pathway (Sarkar et al. 2013). Also, in infected cells with *L. donovani* or *L. major* there is an overexpression of Bcl-xL (Donovan et al. 2009). Our group has also shown that the infection of monocyte-derived dendritic cells with *L*. *mexicana* provokes an overexpression of Bcl-xL as a possible strategy to inhibit apoptosis. (Rodríguez-González et al. 2018). Interestingly, it has been observed that when the genic expression of Bcl-2 or Mcl-1 in *Leishmania donovani*-infected cells is inhibited, the parasite load in the cells and the parasite survival ratio diminish. In addition, it has been observed that in patients with visceral leishmaniasis, the antiapoptotic protein Bcl-2 is overexpressed (Giri et al. 2016; Pandey et al. 2016) (Fig. 4).

## Conclusion

Intracellular pathogens have been subjected to selection pressures that allowed them to develop strategies to prevent or delay apoptosis of the host cell, granting them to reproduce successfully. Nevertheless, once they have reproduced sufficiently, it is necessary for them to release from host cells to invade new cells. One of the best mechanisms to achieve this goal is the induction of apoptosis, since this type of programmed cell death is a noiseless one that can proceed unattended. Pathogens are silently released inside apoptotic bodies, that protect them from the extracellular milieu, and are finally phagocytosed by the neighbor cells in such a way that they do not activate inflammation.

The modulation of apoptosis by intracellular pathogens has been amply analyzed, but still there are many aspects that have not been unveiled. The continuation of these studies is crucial for the development of new strategies and, probably drugs, that could potentially lead to the eradication of the diseases caused by these intracellular pathogens. Over the last few years, it has been observed in studies about the modulation of apoptosis by intracellular pathogens that blocking the parasite capacity to inhibit host cell apoptosis, negatively impacts on the survival of the pathogen, which will hopefully lead to the development of drugs that inactivate pathogen molecules in charge of modulating apoptosis. It is possible that this will aid the development of specific drugs for each pathogen, which will be of great help above all in for the diseases caused by protozoans and fungi, since the drugs now available are nonspecific, have serious side effects and, in addition, many pathogens have started to be resistant to those drugs.

## Data Availability

The data that support the findings of this study are openly available in our laboratory files.
